# Whey Derivatives and Galactooligosaccharides Stimulate the Wound Healing and the Function of Human Keratinocytes through the NF-kB and FOXO-1 Signaling Pathways

**DOI:** 10.3390/nu14142888

**Published:** 2022-07-14

**Authors:** Loredana Bergandi, Tania Flutto, Sabina Valentini, Laura Thedy, Rita Pramotton, Simona Zenato, Francesca Silvagno

**Affiliations:** 1 Department of Oncology, University of Torino, 10126 Torino, Italy; loredana.bergandi@unito.it; 2 Institut Agricole Régional, 11100 Aosta, Italy; t.flutto@iaraosta.it (T.F.); s.valentini@iaraosta.it (S.V.); l.thedy@iaraosta.it (L.T.); r.pramotton@iaraosta.it (R.P.); s.zenato@iaraosta.it (S.Z.)

**Keywords:** whey derivatives, galactooligosaccharides, keratinocyte, differentiation, wound healing, interleukin-8, NF-kB, FOXO-1

## Abstract

Skin repair requires the activation of keratinocytes and is mediated by controlled inflammation and cell migration and proliferation, ending with the regeneration of well-differentiated cell layers. Whey derivatives contain galactooligosaccharides (GOS), which have potential beneficial effects on wound healing due to their activity as toll-like receptor ligands, although their direct nonprebiotic effects in the skin have not yet been described. In this study, we investigated the effects of different whey-derived products and purified GOS on a human keratinocyte cell line. We found that the inflammatory cytokine interleukin-8 (IL-8) was upregulated by nuclear factor kappa B (NF-kB) signaling triggered by whey derivatives and GOS and that wound healing was accelerated by promoting cell migration and the loss of E-cadherin in the absence of epithelial–mesenchymal transition. Interestingly, the treatments enhanced the mitochondrial function in association with the translocation of the Forkhead Box O1 (FOXO-1) transcription factor. Finally, we detected the increased expression of the differentiation markers induced by GOS and whey derivatives. All together, our results show that GOS-containing products can promote wound closure and skin health by direct activity on keratinocyte functions. Among the preparations tested, the fermented compound produced by autochthonous microorganisms was the most active in modulating keratinocyte activity, supporting the biological value of whey derivatives for health.

## 1. Introduction

Galactooligosaccharides (GOS), generally obtained from bovine milk, are galactose polymers with a degree of polymerization of 2–8 and a terminal glucose unit that are synthesized through consecutive transgalactosylation reactions [1]. In addition to their well-documented prebiotic actions on intestinal microbiota, these compounds exert direct, nonprebiotic actions on intestinal barrier function. Indeed, recent studies reported that GOS can attenuate inflammation [2] and enhance immune response in human intestinal epithelial cells [1]. The direct, microbiota-independent effects of oligosaccharides on the intestinal epithelium are due to the direct stimulation of both intestinal epithelial cells [3] and the mucin-producing goblet cells [4]. GOS and other nondigestible oligosaccharides exert their nonprebiotic effects via the activation of Toll-like receptor 4/nuclear factor-kappa B (TLR4/NF-kB) in intestinal epithelial cells [5] and through the modulation of the peroxisome proliferator-activated receptor-γ (PPAR-γ) and NF-kB pathways [6]. The studies investigating the additional properties of GOS have been understandably carried out mainly on the intestinal model, since these preparations are intended as functional food acting on the microflora and microenvironment of the gut [7].

To date, there are no published studies on the direct effects of GOS on skin, notwithstanding the findings that this tissue has the potentiality of benefitting from the activity of prebiotics. Indeed, the complex bidirectional interaction in the skin between resident and pathogen microorganisms on one side and epidermal and immune cell populations on the other side could be influenced by the prebiotic and nonprebiotic effects of several nondigestible oligosaccharides.

The host immune system and the skin microbiota are in constant communication in order to maintain a steady equilibrium [8]. In fact, given the constant exposure to microorganisms, it is important for the host to maintain an adequate cutaneous immune response that adapts it to the occurrence of an infection, as persistent activation against resident skin bacteria could lead to chronic inflammatory disorders [8].

In addition to immune cells, keratinocytes provide an important support in infection and tissue injury through the expression of Toll- and Nod-like receptors that drive the secretion of antimicrobial peptides, chemokines and proinflammatory cytokines [9]. Keratinocytes play therefore a key role in creating both physical and immunologic barriers that protect skin from external threats and that are continuously remodeled during infections and wound repair. Moreover, in response to endogenous RNA released by necrotic cells, skin wounding activates keratinocytes via TLRs to induce inflammation [10]. Both in immune cells [11] and in keratinocytes [12], the activation of TLR-signaling pathways induced by pathogens, resident microorganisms or cell damage leads to NF-kB transactivation, and the consequent secretion of inflammatory cytokines such as interleukin-1 (IL-1), interleukin-6 (IL-6), tumor necrosis factor α (TNFα) and interleukin-8 (IL-8), which initiate the inflammatory immune responses. The healing process requires a controlled inflammation, whereas an exacerbated response can slow the repair process and be harmful. Based on the effects of GOS described in intestinal epithelial cells, it is conceivable that the same nonprebiotic activity could be exerted on keratinocytes.

Under normal physiological conditions, wound healing is a highly ordered biological process that comprises three independent and overlapping phases: hemostasis/inflammation, proliferation and remodeling [13]. In response to skin wounds, proinflammatory cytokines are among the first factors to be produced. They regulate the functions of immune cells in epithelialization [14] and contribute to the epithelialization phase by mobilizing resident stem cells to promote both cellular proliferation and differentiation [9]. Only the controlled and moderate immune responses promote wound healing, as the prevention of infections and accelerated tissue repair occur at normal levels of proinflammatory cytokines but their excessive production is detrimental to normal wound healing. Among the molecular determinants of keratinocyte activation in wound repair, the modulation of cadherins is well-known. Cadherins are a superfamily of transmembrane proteins mediating the intercellular adhesive interactions [15]; E- and P-cadherin are the main molecules involved in the intercellular interactions in the adherens junctions of basal keratinocytes, and they are of crucial importance for the mechanical integrity of the epidermis. E-cadherins are down-regulated by Snail transcription factors during the re-epithelialization of healing wounds [16]. The conditions that decrease E-cadherin expression enhance keratinocyte migration; for example, under hypoxia, E-cadherin expression in the HaCaT keratinocyte cell line was significantly reduced and associated with increased cell migration rates [17]. Additionally, transforming growth factor beta (TGF-β) is a known negative modulator of E-cadherin expression and an inducer of cell migration in the pro-tumoral events known as epithelial–mesenchymal transition [18].

The presence of bioactive GOS has been reported in whey obtained from cheese processing [19,20]; in this material, GOS originate partly from the bovine mammary gland and partly from the activity of microorganisms expressing β-galactosidase that are employed in cheese making. In addition to GOS among the by-products of cheese making, other molecules possess bioactive properties. Several studies have reported that the action of lactic acid bacteria on dairy products may contribute to the formation of free fatty acids such as butyrate [21], which have attracted much attention as novel beneficial functional lipids [22,23]. GOS are counted among the natural products with beneficial activity for human health. In fact, many natural compounds are investigated for their antiangiogenic, anti-inflammatory and antioxidant effects [24,25,26] in pathologies such as diabetes and cancer [27,28,29]. Whey derivatives and GOS are mainly studied in the intestine, but novel properties remain to be discovered.

The aim of this study was to investigate the effects of different preparations of nondigestible oligosaccharides obtained from whey processing on a human keratinocyte cell line. We tested the biological activities of GOS relevant for wound healing: the production of inflammatory cytokines, cell migration, energy metabolism and differentiation of HaCaT cells. Moreover, we assayed the molecular signaling triggered by the whey derivatives and GOS in keratinocytes. All together, our results show that these compounds can promote wound closure.

## 2. Materials and Methods

### 2.1. Cells and Reagents

The immortalized human epidermal keratinocyte HaCaT cell line was cultured in Dulbecco’s modified Eagle’s medium (DMEM) high glucose (4500 mg/L), supplemented with 10% fetal bovine serum (FBS) and 1% antibiotics (penicillin-streptomycin) at 37 °C in a humidified atmosphere containing 5% carbon dioxide (CO_2_). Unless otherwise specified, reagents were purchased from Sigma-Aldrich (Milan, Italy), whereas plastic ware was from Falcon (Becton Dickinson, Franklin Lakes, NJ, USA). *Mycoplasma* spp. contamination was assessed by polymerase chain reaction (PCR) every week; contaminated cells were discharged, with the only exception for the experiments performed on mycoplasma-infected cells, used to mimic real infection.

### 2.2. Preparation of Whey Derivatives

First, the process of clarification by thermocalcic precipitation, followed by microfiltration, was used to remove the non-centrifugable residual lipids present in whey, according to the method of Fauquant et al. [30] and modified by Pereira et al. [31]. After ultrafiltration using a crossflow system (Cogent µ-scale, Merck Millipore, Milan, Italy) with a membrane of 10 kDa molecular weight cut-off, the obtained whey permeate (perm) underwent controlled fermentation by newly characterized autochthonous microorganisms isolated in the Aosta Valley (Italy): *Lactobacillus delbrueckii* ssp. unknown MF-20/7A/24, deposited with the BCCM/LGM Bacteria Collection (Gent University, Belgium; patent filing N. LMG P-31789). The bacterial culture was inoculated 1:100 *v*/*v* to brew the whey permeate, which was previously pasteurized in autoclave. The fermentation was conducted at 37 °C for 24 h, after which the fermented product (ferm) was subjected again to autoclaving and microfiltration to remove microbial residues. The obtained preparations (perm and ferm) were characterized in terms of milk solid non-fat components using milk AOAC (Association of Official Analytical Chemists) protocols adapted to whey, whereas the carbohydrate fraction (lactose, glucose and galactose) was quantified using a specific enzymatic kit (Megazyme, Bray, Ireland). The indirect measure of the amount of GOS available before and after fermentation was obtained from the differences between the total solids and the carbohydrate components, minerals and ash. Butyrate was quantified by an solid-phase microextraction followed by gas chromatography-mass spectrometry (SPME-GC/MS) chromatographic method, modified from Pan et al. [32]. The GC oven temperature was held at 40 °C for 4 min and increased from 40 to 60 °C at a rate of 5 °C/min, 60 to 120 °C (6 °C/min) and 120 to 230 °C (10 °C/min). The GC–MS transfer line was maintained at 250 °C. The mass spectra were collected in electron ionization mode. The comparison of perm and ferm composition revealed that the GOS components were not enriched after fermentation, changing from 3.29 (% *w*/*v*) to 2.93 (% *w*/*v*); instead, the concentration of butyrate in perm was 0.00153 g/L and following the fermentation increased to a maximum of 0.00514 g/L, equivalent to 0.52 µM and 1.75 µM, respectively.

### 2.3. Cell Treatments

HaCaT cells were seeded, and on the second day of culture, the medium was removed and replaced with fresh 10% FBS medium containing 3% of commercial purified GOS (guaranteed reagent grade of GOS with purity equal to 74.7% of the anhydrous substance was purchased from Galeno Srl, Comeana, Italy) or 3% of fermentate or 3% of permeate diluted in distilled water or butyrate 1.75 µM (corresponding to the butyrate quantity in fermentate preparation). The concentration of the tested compounds was chosen by evaluating which amount of whey derivatives could be used in a topical preparation in vivo in future studies. In all experiments, the medium was refreshed every day to avoid fluctuations in the concentrations of the tested compounds. Untreated cells were used as negative control. For NF-kB and FOXO-1 analysis, keratinocytes were treated with the TLR ligand lipopolysaccharide from *Escherichia coli* O111:B4 (LPS) (20 μg/mL) for 24 h.

### 2.4. Cell Proliferation Assay

The effects of different preparations of non-digestible oligosaccharides on the growth of HaCaT cell line were determined by the colorimetric measurement of cell numbers by crystal violet staining. An amount of 750 cells was seeded in each well of 96-multiwell plates, and at the end of the incubation period, the cells were fixed for 15 min with 11% glutaraldehyde; then, the plates were washed three times, air-dried and stained for 20 min with a 0.1% crystal violet solution. The absorbance was determined at 595 nm [33] after washing, drying and solubilization of the bound dye with a 10% acetic acid solution. The data collected from six wells were averaged for each experimental condition, and each experiment was repeated three times. A sample was considered cytotoxic if cell proliferation rate was ≤70% compared with the untreated. Sodium dodecyl sulfate (SDS) 0.002% was used as positive control.

### 2.5. Cell Viability Assays

Cell viability was assessed using the (3-[4,5-dimethylthiazol-2-yl]-2,5-diphenyltetrazoliumbromide) MTT assay [34]. On day 1, 5000 cells/well were seeded into 96-well plates in 100 µL culture medium, and on day 2 compounds were added. After 48 h of treatments, MTT (0.5 mg/mL) was added to each well, and the supernatants were removed after 4 h of incubation at 37 °C. The absorbance values at 570 and 630 nm were determined using a microplate reader Victor 3 from PerkinElmer Life Sciences (Waltham, MA, USA) after the solubilization of the formazan crystals using 100 µL dimethyl sulfoxide (DMSO). In addition, after the incubation period, the cells grown on 96-multiwell plates were stained for 1 h at 37 °C in culture medium containing Neutral Red solution, washed three times with phosphate-buffered saline solution (PBS) and rinsed with stop buffer (4.02 g trisodium citrate in 153 mL water (H_2_O), 0.8 mL hydrochloric acid (HCl) 0.1 N in 86 mL H_2_O and 25 mL of 95% *v*/*v* methanol). The absorbance was read at 540 nm, and the cell viability was evaluated by measuring the percentage of cells stained with neutral red dye, as previously reported [35].

The results were expressed as percentage of viable cells in each experimental condition versus untreated cells. The data collected from six wells were averaged for each experimental condition, and each experiment was repeated three times. A sample was considered cytotoxic if the cell viability was ≤70% compared with the untreated. SDS 0.002% was used as positive control.

### 2.6. Wound Healing Assay

Cells were seeded in a 24-well plate, and when they reached confluency, they were starved overnight. A wound line was generated with a sterile pipette tip, followed by treatments for 24 h. TGF-β (10 ng/mL) was used as positive control. Pictures were taken with a digital camera under bright field illumination using a light microscope at 20× magnification. The central part of each well was measured for wound area using ImageJ software (ImageJ version 1.29, Sun Microsystems Inc., Palo Alto, CA, USA). The measurements were then converted into a percentage of wound closure: 100 − [(area at t24/area at t0) × 100] [36].

### 2.7. Real-Time Polymerase Chain Reaction (qRT-PCR)

HaCaT cells were seeded on 6 multiwell plates, and after the incubation period, cells were washed with PBS, and total RNA was extracted with TRIzol^®^ (Invitrogen, Thermo Fisher Scientific, Waltham, MA, USA). One μg of total RNA was reversely transcribed into cDNA, in a final volume of 20 μL, using the iScriptTM cDNA Synthesis Kit (Bio-Rad, Hercules, CA, USA) according to the manufacturer’s instructions. The RT-PCR primers were designed with NCBI/Primer-BLAST, synthesized by Sigma Aldrich (Milan, Italy) [37]. Quantitative PCR was carried out in a final volume of 20 μL using the iTaqTM Universal SYBR^®^ Green Supermix (Bio-Rad, Hercules, CA, USA) with specific primers for the quantitation of the following human genes: E-cadherin (E-cadherin, fwd 5′-TACGCCTGGGACTCCACCTA-3′, rev 5′-CCAGAAACGGAGGCCTGAT-3′), vimentin (vimentin, fwd 5′-AGGAAATGGCTCGTCACCTTCGTGAATA-3′, rev 5′-GGAGTGTCGGTTGTTAAGAACTAGAGCT-3′), involucrin (involucrin, fwd 5′-CTGCCTCAGCCTTACTGTGA-3′, rev 5′-GGAGGAGGAACAGTCTTGAGG-3′), Keratin 1 (KRT1, fwd 5′-ATTTCTGAGCTGAATCGTGTGATC-3′, rev 5′-CTTGGCATCCTTGAGGGCATT-3′), keratin 10 (KRT10, fwd 5′-TGATGTGAATGTGGAAATGAATGC-3′, rev 5′-GTAGTCAGTTCCTTGCTCTTTTCA-3′), cytochrome c oxidase subunit 2 (COX II, fwd 5′-TCTGGTCAGCCCAACTCTCT-3′, rev 5′-CCTGTGATCCACCAGAAGGT-3′), mitochondrial adenosine 5′-triphosphate (ATP) synthase F0 subunit 6 (MT-ATP6, fwd 5′-CCAATAGCCCTGGCCGTAC-3′, rev 5′-CGCTTCCAATTAGGTGCATGA-3′), interleukin-8 (IL-8, fwd 5′-GGAGAAGTTTTTGAAGAGGGCTGA-3′, rev 5′-TGCTTGAAGTTTCACTGGCATCTT-3′) and ribosomal subunit protein (S14, fwd 5′-AGGTGCAAGGAGCTGGGTAT-3′, and rev 5′-TCCAGGGGTCTTGGTCCTATTT-3′). The real-time PCR parameters were as follows: 1 cycle of denaturation at 95 °C for 30 s, 45 cycles of amplification including denaturation at 95 °C for 5 s and annealing/extension at 60 °C for 30 s. Applying the 2-ΔΔCT method, the quantification of PCR gene product was expressed in arbitrary units, using the Bio-Rad Software Gene Expression Quantitation (Bio-Rad Laboratories, Hercules, CA, USA). The housekeeping gene ribosomal subunit protein S14 was used as internal control. The similar PCR efficiency and high linearity amplification plots (*r* > 0.98) of the analyzed transcripts confirmed that the expression of each gene could be directly compared. Melt curve analysis confirmed the specificity of PCRs.

### 2.8. Western Blot Analysis

HaCaT cells were seeded on 6 multiwell plates, and after the incubation period, subcellular fractionation and western blotting analyses of cytosolic, nuclear and mitochondrial proteins were carried out as previously described [38]. An amount of 50 μg of extracts was subjected to 10% or 12% sodium dodecyl-sulfate polyacrylamide gel electrophoresis (SDS-PAGE) and transferred to polyvinylidene fluoride (PVDF) membrane. The blots were blocked with 5% non-fat milk in PBS at room temperature (RT) for 1 h and incubated overnight with the following antibodies: rabbit anti-NF-kB p65 (sc-109, Santa Cruz Biotechnology Inc., Santa Cruz, CA, USA) and mouse anti-forkhead box O1 (FOXO-1) (diluted 1:1000 *v*/*v* in 1% PBS-BSA, sc-374427, Santa Cruz Biotechnology Inc., Santa Cruz, CA, USA). The mouse anti-actin antibody (sc-8432, Santa Cruz Biotechnology Inc., Santa Cruz, CA, USA) (diluted 1:1000 *v*/*v* in 1% PBS-bovine serum albumin (BSA)) and the mouse anti-TATA binding protein (TBP) (diluted 1:500 *v*/*v* in 1% PBS-BSA, sc-421, Santa Cruz Biotechnology Inc., Santa Cruz, CA, USA) were used to check, respectively, the equal cytosolic and nuclear protein loading. Mouse anti-voltage-dependent anion channel (VDAC) antibody (anti-porin 31HL, Calbiochem, Merck S.p.a., Milan, Italy) was diluted 1:500 *v*/*v* in 1% PBS-BSA and used to confirm the quality of the mitochondrial purification. The same membranes were used to detect the proteins of interest and the loading controls. After an overnight incubation, the membrane was washed with 0.1% *v*/*v* PBS-Tween and subjected for 1 h to a peroxidase-conjugated anti-mouse or anti-rabbit secondary antibody (diluted 1:5000 *v*/*v* in 5% *w*/*v* PBS-Tween with milk, Bio-Rad Laboratories, Hercules, CA, USA). The membrane was washed again with PBS-Tween, and proteins were detected and quantified by ChemiDocTM MP System (Bio-Rad Laboratories, Hercules, CA, USA). Densitometric analysis was carried out using ImageJ software (ImageJ version 1.29, Sun Microsystems Inc., Palo Alto, CA, USA)

### 2.9. Statistical Analysis

The data were expressed as the mean ± standard error (SEM) of three independent experiments. The statistical analysis of the data was performed using ANOVA with Tukey’s post hoc correction, using GraphPad Prism 5.0 (GraphPad Software, Inc., San Diego, CA, USA). Values for *p* < 0.05 were considered significant and indicated.

## 3. Results

### 3.1. GOS Induce the Production of IL-8 through NF-kB Signaling

**Figure 1:**
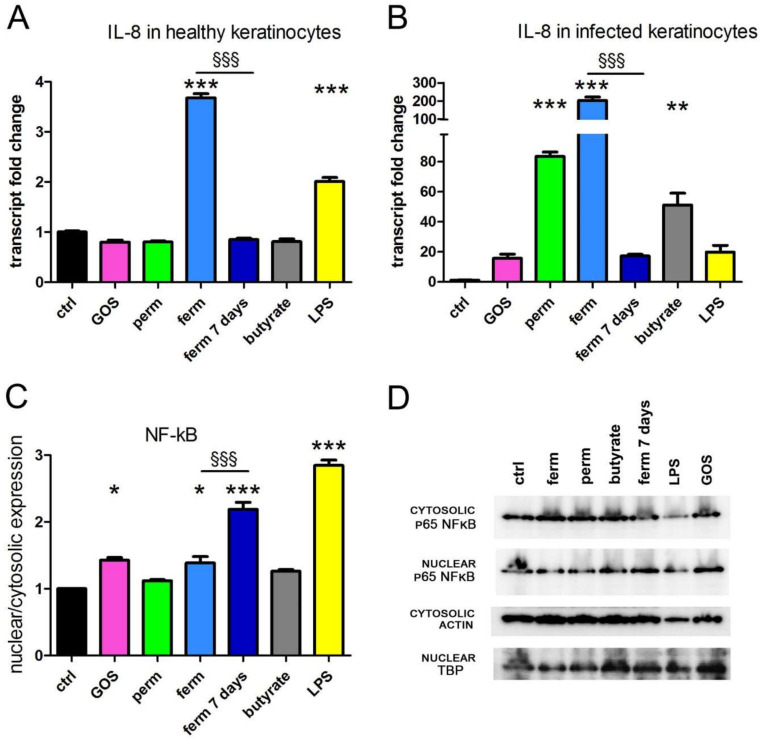
The fermented product induces IL-8 transcription. HaCaT cells were treated for 24 h as indicated; (**A,B**) the levels of IL-8 transcript were assessed by real-time PCR. (**C**) NF-kB protein expression was detected by western blot from the cytosolic and nuclear extracts of healthy cells. Actin and TBP expression were used as loading control. Bands were quantified relative to loading control, and the translocation from cytosol to the nuclear compartment was evaluated as the ratio of nuclear vs. cytosolic expression. (**D**) A representative blot is shown, and the whole blots are displayed in Supplementary Materials Figure S1. The data represent the means ± SEM of three independent experiments. * *p* < 0.05, ** *p* < 0.01 and *** *p* < 0.001 compared with the control (ctrl); §§§ *p* < 0.001. IL-8, interleukin-8; PCR, polymerase chain reaction; NF-kB, nuclear factor kappa B; TBP, TATA-Box Binding Protein; SEM, standard error; GOS, galactooligosaccharides; ctrl, control; perm, permeate; ferm, fermented product; LPS, lipopolysaccharide from *Escherichia coli* O111:B4.

GOS and whey derivatives, as both permeate and fermented ingredient, were tested for their ability to influence wound healing. During the inflammatory phase of wound repair, keratinocytes upregulate IL-8 [39], which is involved in immune cell recruitment [40]. Therefore, we investigated IL-8 expression by the real-time PCR analysis of HaCaT cells treated for 24 h with purified GOS and whey derivatives, both in healthy cells and in a model of mycoplasma-infected cells. The latter represents an in vitro model of infected skin [41,42]. Moreover, the biological activity of butyrate was tested because this small fatty acid is enriched in the ferm product. To this purpose, butyrate was used at the same concentration calculated in the fermented product preparation. As shown in Figure 1A,B, the fermented product induced a striking increase of IL-8 transcription in both models, whereas permeate and butyrate were very effective only in infected cells. After the treatment of infected cells with purified GOS and after prolonged incubation with fermented products, the increase of IL-8 transcription was evident (20-fold increase), although not significant on ANOVA. In these experimental sets, LPS was used as a known infective stimulant of cytokine production; indeed, it induced IL-8 expression, with a twofold increase in healthy cells and a twenty-fold increase in infected HaCaT cells. In inflammation, the production of cytokines is controlled by the NF-kB signaling pathway; therefore, further experiments sought to investigate whether purified GOS and whey derivatives triggered NF-kB activation. We analyzed the nuclear translocation of p65 protein in healthy cells only, as NF-kB is potently stimulated by mycoplasma infection [43,44]. As quantified in Figure 1C, the nuclear import of NF-kB was enhanced after treatment with purified GOS, with the fermented product (both short and prolonged exposure), and of course with LPS, a known inducer of NF-kB activation. All together, our experimental results demonstrate that the fermented product is the most effective inducer of the NF-kB signaling pathway and a potent stimulator of IL-8 production in human keratinocytes.

### 3.2. Whey Derivatives and GOS Increase the Motility of HaCaT Cells

**Figure 2:**
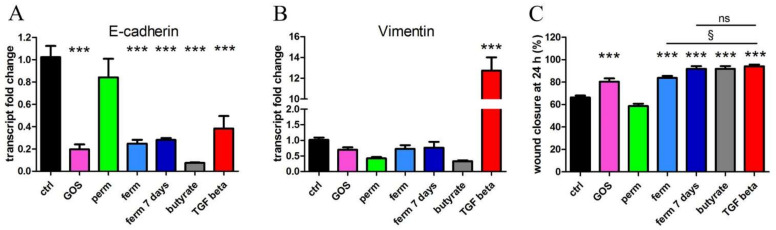
GOS and whey derivatives increase wound healing. HaCaT cells were treated for 24 h with purified GOS, whey permeate (perm), fermented product (ferm), butyrate and TGF-β (TGF beta) or were exposed to fermented material for 7 days. Transcription of E-cadherin (**A**) and vimentin (**B**) was evaluated by real-time PCR. (**C**) After the same treatments, wound closure was assessed by wound healing assay. The data represent the means ± SEM of three independent experiments. Images of wound healing assay are displayed in Supplementary Materials Figure S2. *** *p* < 0.001 compared with the control (ctrl); § *p* < 0.05; ns: not significant; TGF, transforming growth factor.

In the wound-healing process, the activation and recruitment of keratinocytes to the damaged site requires the loss of the tight connections between keratinocytes, followed by their migration to fill the wound; based on these considerations, we first measured the transcription of E-cadherin in HaCaT cells treated with permeate and fermented product, comparing their efficacy with the effects of a purified GOS preparation. We also tested butyrate. The cells were treated for 24 h, and then transcript levels of E-cadherin were measured. As shown in Figure 2A, E-cadherin was downregulated by GOS and the whey-derived fermented product, whereas the permeate was not active on the modulation of gene expression. Butyrate demonstrated the same effect shown by the GOS and the whey-derived fermented product, as well as the prolonged incubation for 7 days with fermented material, which confirmed the persistent effect of this preparation. In addition to its involvement in intercellular attachment, E-cadherin is downregulated in epithelial–mesenchymal transition (EMT), an initial step of neoplastic transformation predisposing to metastasis formation. To check whether EMT was induced by the treatments, we measured vimentin transcription, which was not modulated, as shown in Figure 2B; therefore, we could rule out this unwanted effect. As a control of a treatment that induces EMT, we verified that TGF-β potently increased vimentin and decreased E-cadherin. Next, cell migration was tested by wound-healing assay. As quantified in Figure 2C, purified GOS induced a significant accelerated wound closure, as well as the fermented product, and the treatments with butyrate and TGF-β were even more effective; the latter was used again as a known stimulator of cell motility. Interestingly, the effect of the prolonged exposure to fermented product was more potent than short exposure, as the closure was identical after 7 days of fermentation and after the TGF-β treatments.

All together, these results demonstrate that GOS, whey-derived fermented preparation and butyrate can promote the steps necessary for wound healing, which are the loss of epithelial intercellular adhesive interactions mediated by cadherins and the keratinocyte migration, but the harmful EMT is avoided.

### 3.3. GOS Enhance Mitochondrial Activity in Association with Mitochondrial Export of FOXO-1

**Figure 3:**
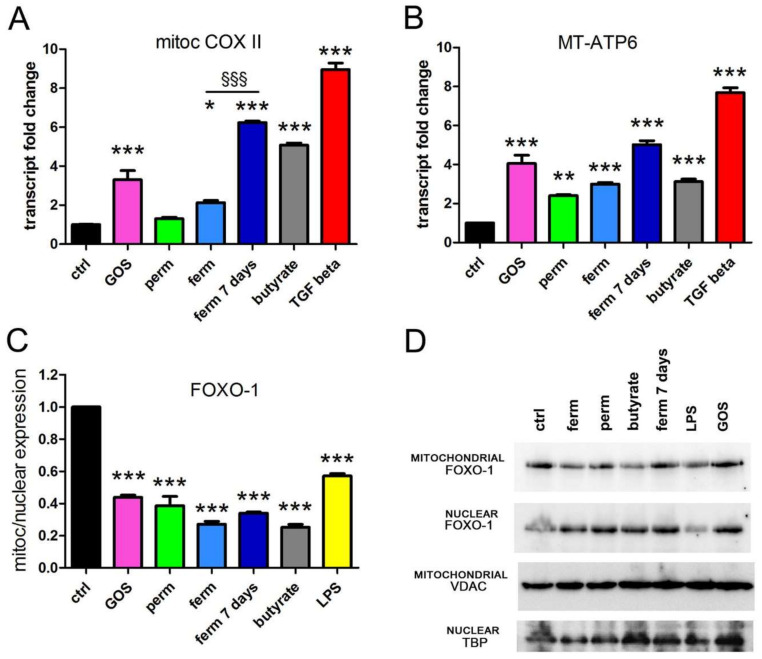
GOS and whey derivatives induce the transcription of respiratory subunits and promote FOXO-1 translocation. HaCaT cells were treated for 24 h as indicated, and the levels of mitochondrial transcripts were assessed by real-time PCR (**A**,**B**). FOXO-1 protein expression was detected by western blot from nuclear and mitochondrial extracts. VDAC and TBP expression was used as loading control. (**C**) Bands were quantified relative to loading control, and the translocation from the mitochondrial to the nuclear compartment was evaluated as the ratio of mitochondrial vs. nuclear expression. (**D**) A representative blot is shown, the same membrane displayed in Figure 1, and the whole blots are available in Supplementary Materials Figure S1. The data represent the means ± SEM of three independent experiments. * *p* < 0.05, ** *p* < 0.01 and *** *p* < 0.001 compared to the control (ctrl); §§§ *p* < 0.001. FOXO-1, Forkhead Box O1; VDAC, voltage-dependent anion channel; mitoc COX II, mitochondrial cytochrome c oxidase subunit 2; MT-ATP6, mitochondrial adenosine 5′-triphosphate (ATP) synthase F0 subunit 6.

The enhanced cell migration observed after treatment with different sources of GOS should be associated with an increased energy expenditure. To verify if this was the case, we tested the activation of the main mechanism of ATP production, mitochondrial respiration coupled with oxidative phosphorylation (OXPHOS). The evaluation of the mitochondrial transcription of one subunit of complex IV (mitochondrial COX II) and one subunit of ATP synthase (mitochondrial MT-ATP6) was carried out by real-time PCR analysis. Figure 3 shows that purified GOS, fermented product and butyrate induced the transcription of the respiratory complex and ATP synthase, whereas permeate was less effective. In this experimental set, the treatment with TGF-β was considered a positive control since the cytokine is a respiratory modulator [45]. Next, we wondered if any factor, and if so, which, could modulate the mitochondrial metabolism in response to GOS stimulation. In human keratinocytes, the mammalian FOXO-1 can localize in both the mitochondrial and nuclear compartments, exerting transcriptional activity in both sites, and its shuttling is responsive to extracellular stimulation [46]. Whereas as nuclear factor FOXO-1 regulates key markers of keratinocyte differentiation [47], it can act as a transcription repressor in mitochondria [46]. Based on this evidence, this factor seemed a good candidate as the mediator of the differentiating and metabolic effects of GOS. Indeed, the analysis of intracellular localization showed that all experimental conditions stimulated the transfer of the transcription factor from mitochondria to nuclei as the ratio of mitochondrial to nuclear expression decreased. The results of the protein analysis and quantification are shown in Figure 3C,D.

### 3.4. Whey Derivatives and GOS Increase the Differentiation of HaCaT Cells

**Figure 4:**
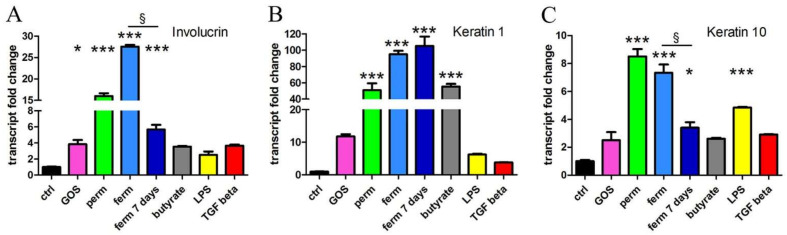
GOS and whey derivatives induce the transcription of differentiation markers ((**A**) involucrin, (**B**) keratin 1, (**C**) keratin 10). After 24 h of treatment with purified GOS, whey permeate (perm), fermented product (ferm), butyrate, LPS and TGF-β or after exposure to fermented material for 7 days, the transcript levels were measured with real-time PCR. The data represent the means ± SEM of three independent experiments. * *p* < 0.05 and *** *p* < 0.001 compared to the control (ctrl); § *p* < 0.05.

Epithelialization is essential in the wound-healing process, which requires restoring an intact keratinocyte layer through the differentiation and production of molecules necessary for barrier formation. Interestingly, FOXO-1 regulates key markers of keratinocyte differentiation such as involucrin [47]. We evaluated differentiation by real-time PCR analysis of three genes upregulated in differentiated keratinocytes. As shown in Figure 4, whey derivatives strongly induced the transcription of involucrin and of keratins 1 and 10, both in the permeate and fermented products. The prolonged treatment with fermented material led to a lighter induction of involucrin and keratin 10. The purified GOS were much less effective: Although involucrin and keratin 1 expression were fairly augmented by GOS treatment, the statistical analysis of all treatments comprehensive of whey derivatives made the increase triggered by GOS significant only for involucrin. The differentiating property of butyrate was particularly evident in terms of the induction of keratin 1 expression.

Because terminal differentiation in epidermal keratinocytes in vivo is associated with death, we verified whether the treatments could be both differentiating and toxic. Whey permeate, the fermented ingredient, the purified GOS preparation and butyrate were tested to exclude the cytotoxicity and modulation of proliferation. We found that they did not affect the viability and proliferation of HaCaT cells (Supplementary Materials Figure S3).

## 4. Discussion

In this study, we demonstrated for the first time the direct effects of purified GOS and whey derivatives on human keratinocytes and revealed the potential beneficial effects of GOS on human skin. To date, the few studies have described only the indirect, gut- mediated activity of dietary GOS on skin health [48,49]. The results of our experiments showed that several steps of wound healing can benefit from the direct administration of GOS to keratinocytes. Indeed, we discovered that GOS stimulate the inflammatory, migratory and differentiation events that are necessary to restore the intact skin barrier.

We tested different sources of GOS, both a purified preparation and the results of fractionation of whey obtained from cheese processing with autochthonous microorganisms. The two analyzed materials, permeate and fermented product, were different not in the quantity but rather in the quality of the GOS and biological activity; as we did not find evident differences in weight composition, we believe that the fermentation carried out by lactobacilli must have impacted the polymerization degree and butyrate synthesis. Because of this modified composition, in our experimental model, the fermented product was more active than permeate and even more active than purified GOS. Because we demonstrated the biological activity of butyrate, we conclude that the superiority of fermented product can be ascribed at least in part to the presence of butyrate in the preparation. We cannot rule out that in our preparation, other yet untested molecules could have an impact on wound healing, and further investigation is warranted. For example, whey-derived peptides have recognized biological activity [50]. The comparison carried out in this study supports the biological value of whey derivatives for health. In particular, the byproducts of cheese fabrication are intensely studied with recycling aims, in the attempt of both reducing the high pollutant load of dairy industry and finding a novel nutraceutical utilization; the latter is justified by the presence of organic and inorganic nutrients that make this residue a potential source for transformation into high-added-value products. Based on these considerations, the results of this study are particularly valuable in terms of eco-sustainable alternative uses of whey.

Considering the results as a whole, we demonstrated that GOS and whey derivatives are beneficial during several steps of wound closure, starting from initial inflammation and ending with differentiated epithelial layering. First, we discovered that the inflammatory response of keratinocytes was enhanced by different sources of GOS through the induction of IL-8 transcription, especially in keratinocytes exposed to infection; this cytokine is crucial for immune recruitment in the initial phase of wound healing [51], and the results obtained in the mycoplasma-infected cells demonstrate the potential efficacy of GOS for infected wounds. The fermented product was the only trigger of IL-8 overexpression in healthy cells and the most potent inducer in infected keratinocytes, but the prolonged treatment did not maintain the stimulatory effect; this observation is quite relevant and suggests that the inflammation triggered by GOS is optimal because it is reversible, and it is an acute defense mechanism that does not evolve into a chronic inflammatory response [52]. As reasonably expected, we verified that the cytokine induction was NF-kB mediated, and the persistence of this signaling activation after long treatment suggests that under long-term stimulation, some negative feedback mechanism must control IL-8 expression.

It was previously reported that IL-8 significantly increases the migration of HaCaT keratinocytes [53]. Interestingly, upon stimulation with purified GOS and fermented product, we observed that the cell motility was augmented, together with the decrease of E-cadherin, thus reproducing the loss of intercellular junctions and the increased keratinocyte migration required to fill the wound. As a positive control of the wound-healing assay, we verified that TGF-β promoted keratinocyte migration, as previously reported [54]. Importantly, whereas in our experimental set, TGF-β was a potent inducer of EMT, measured as E-cadherin decrease and vimentin increase, the stimulation triggered by GOS did not provoke the transition, which would be a potentially harmful transformation.

Cell movement requires energy expenditure, and this aspect was influenced by the different preparations of GOS that potentiated the expression of the mitochondrial respiratory elements. It was previously reported that in wound healing, the migration is promoted by increased energy metabolism [55], whereas the alterations in bioenergy function hamper cell migration [56]. The results of this study demonstrated that the components of OXPHOS are upregulated by GOS, confirming the central role of energy production in the cell migration necessary for wound healing. For the first time, this study demonstrates that GOS can sustain cell migration in healing through the mechanism of enhanced energy production, mediated by FOXO-1 translocation. By shuttling from mitochondria to nuclei, on one hand, the transcriptional factor enhances mitochondrial activity; on the other hand, it modulates specific genomic transcription. FOXO-1 has been extensively investigated in adipocytes and in keratinocytes as well. In the mitochondrial matrix of white/beige adipocytes, the phosphorylated isoform of FOXO-1 mitigates the expression of the oxidative phosphorylation subunits encoded by the mitochondrial DNA and determines the transcription of the mitochondrial anti-stress response [57]. Alternatively, nuclear FOXO-1 promotes the transcription of several tissue-specific genes, codifying for adipose triglyceride lipase and antioxidant enzymes in adipocytes. Moreover, FOXO-1 inhibits the expression of PPAR-γ [57]. Keratinocytes are also protected from oxidative stress by FOXO-1, which activates antioxidant defense and DNA repair enzymes [58,59]. The up-regulation of the keratinocyte differentiation markers keratin 1, 10 and involucrin is FOXO-1-dependent in keratinocytes infected by *P. gingivalis* [47]. Moreover, this transcription factor regulates several genes in the keratinocytes participating in wound repair and thus having a crucial role in wound healing. For example, FOXO-1 induces TGF-β [58] and also regulates the expression of integrins-β6 and -α3 [58], needed for keratinocyte migration [60]. Furthermore, several studies have described that the reactive oxygen species (ROS)-mediated regulation of FOXO-1 activity in cellular systems relies on multiple molecular mechanisms. During adipocyte differentiation, the transient increase of ROS is associated with the upregulation of FOXO-1, and notably, in endothelial cells and follicular granulosa cells, the oxidative stress triggers nuclear translocation of FOXO-1 [61,62]. This feedback mechanism is necessary for boosting the antioxidant response via the upregulation of antioxidant enzymes to avoid detrimental oxidative stress [63] as ROS production is tightly balanced during adipogenic differentiation.

In this study, we demonstrated that GOS are able to activate NF-kB, probably through TLRs, as reported for other cell systems [1,64]. NF-kB is a central hub in ROS homeostasis since NF-kB and ROS signaling are strictly linked and stimulate each other; therefore, it is not surprising that under the same experimental conditions, we could detect both the nuclear translocation of NF-kB and the transfer of FOXO-1 from the mitochondrial to the nuclear compartment, leading to the conclusion that this combined translocation is an additional mechanism by which GOS support the inflammatory and migratory properties of keratinocytes.

Interestingly, and again, in line with all the novel observations of this study, GOS were able to enhance differentiation of HaCaT cells. Like the other observed effects, the fermented product was the most effective source of GOS in inducing differentiation, although the prolonged incubation with fermented product was not always able to maintain the same enhanced differentiation as the short stimulation. In fact, both involucrin and keratin 10 expression were less evident after 7 days of treatment, although they were still higher than control, possibly due to a feedback mechanism to check the excessive production of these proteins. Considering all the results obtained when cells were treated for 7 days, we can conclude that the prolonged exposure to GOS and whey derivatives is beneficial to the wound-healing process because of the transient induction of inflammatory cytokines, the stable stimulation of migration and mitochondrial energy production and the correct production of proteins necessary for the restoration of barrier function in differentiated cell layers.

Until now, the beneficial effects of GOS on skin health were described as indirect and intestine mediated, and these molecules were used in prebiotic or symbiotic preparations. The results of this study shed some light on novel properties of GOS and suggest a different direct utilization on the skin, for example by topical application. Further studies in vivo are necessary to confirm and further investigate our original observations.

## 5. Conclusions

**Figure 5:**
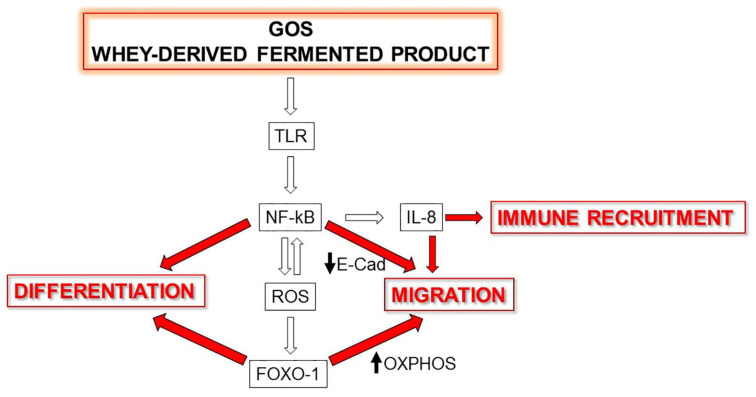
A working model of the molecular mechanisms underlying the effects exerted by GOS on wound healing. TLR, Toll-like receptor; ROS, reactive oxygen species; E-Cad, E-cadherin; OXPHOS, oxidative phosphorylation. Black arrows indicate an increase or a decrease.

In this study, we investigated the direct effect of GOS on the human keratinocyte properties relevant in the wound-healing process. The beneficial reversible inflammatory response, mitochondrial respiration, cell migration and differentiation were all stimulated by several sources of GOS, and we have revealed some insightful details of the molecular mechanisms involved. In fact, for the first time, we demonstrated that the nuclear import of NF-kB and the mitochondrial-to-nuclear translocation of FOXO-1 can be responsible for the biological effects of GOS. A schematic representation of conclusions reached by this study is proposed in Figure 5.

## 6. Patents

Italian patent application no. 102021000011006, 30 April 2021.

## Data Availability

The data presented in this study are available within the article.

## Supplementary Materials

The following supporting information can be downloaded at: https://www.mdpi.com/article/10.3390/nu14142888/s1, Figure S1: Western blot assay of nuclear and cytosolic NF-kB, nuclear and mitochondrial FOXO-1, and loading controls for nuclear, mitochondrial and cytosolic fractions. Figure S2: Pictures of cells used for wound healing assay were taken after scratch (T0) and after 24 h of treatment (T24). Figure S3: GOS and whey derivatives do not affect viability and proliferation of HaCaT cells. After two days of treatment, cells were analyzed by MTT (A) and neutral red assay (B) to exclude cytotoxicity, and proliferation was assessed by crystal violet staining (C). Positive controls for toxicity is represented by 0.002% SDS. The data represent the means ± SEM of three independent experiments. *** *p* < 0.001 compared to the control.
