# Supplementary material for: Whey Derivatives and Galactooligosaccharides Stimulate the Wound Healing and the Function of Human Keratinocytes through the NF-kB and FOXO-1 Signaling Pathways
Source: Nutrients. 2022 Jul 14;14(14):2888. doi: 10.3390/nu14142888 (PMC9319648; doi:10.3390/nu14142888)
Supplement: Supplementary file 1 [file nutrients-14-02888-s001.zip › nutrients-1809820-supplementary.pdf]

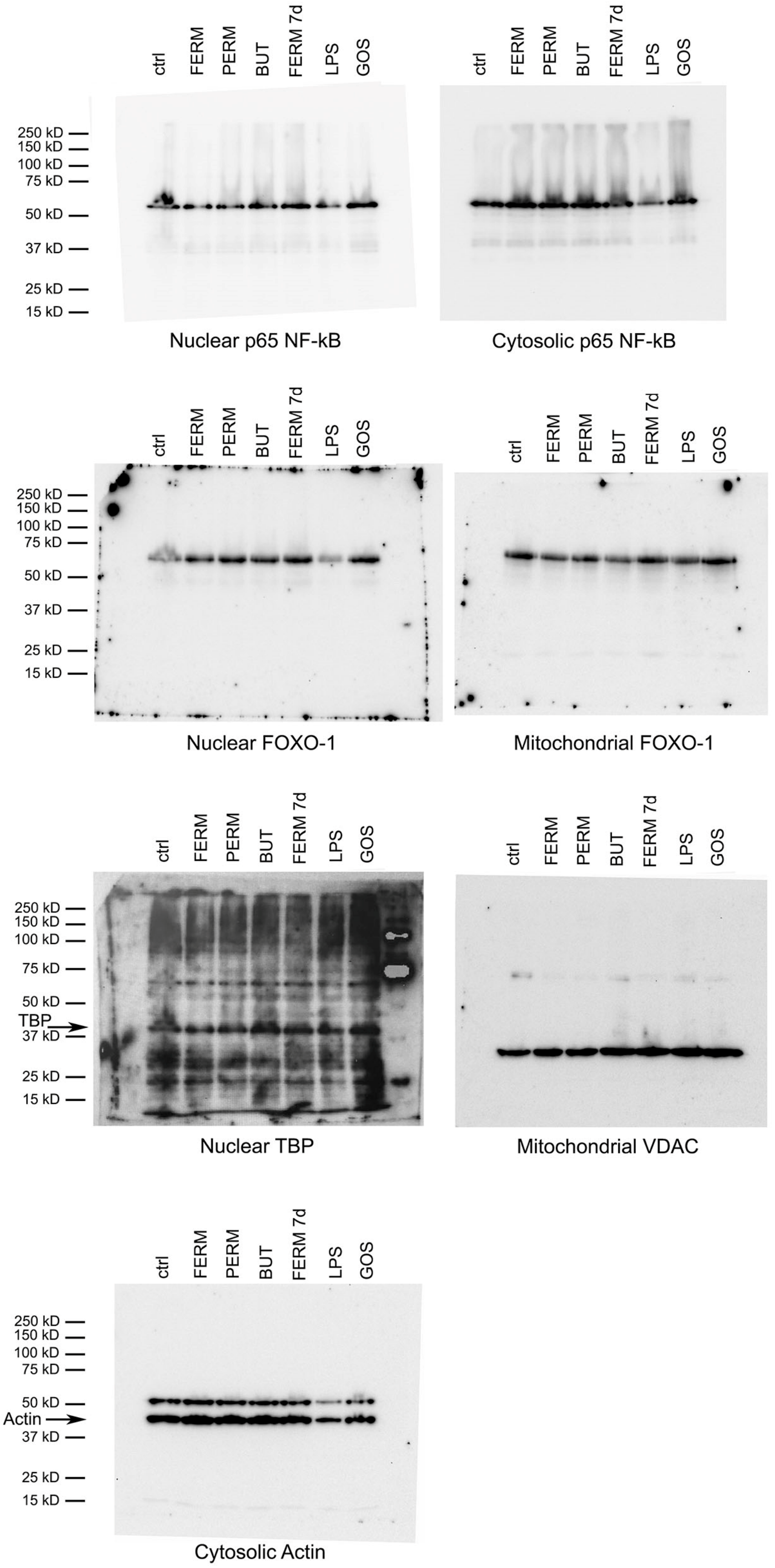

Figure S1. Western blot assay of nuclear and cytosolic NF-kB, nuclear and mitochondrial FOXO-1, and loading controls for nuclear, mitochondrial and cytosolic fractions. CTRL, control; PERM, permeate; FERM, fermented product; BUT, butyrate; LPS, lipopolysaccharide from *Escherichia coli* O111:B4; GOS, galactooligosaccharides; NF-kB, nuclear factor kappa B; FOXO-1, Forkhead Box O1; TBP, TATA binding protein; VDAC, voltage-dependent anion channel.

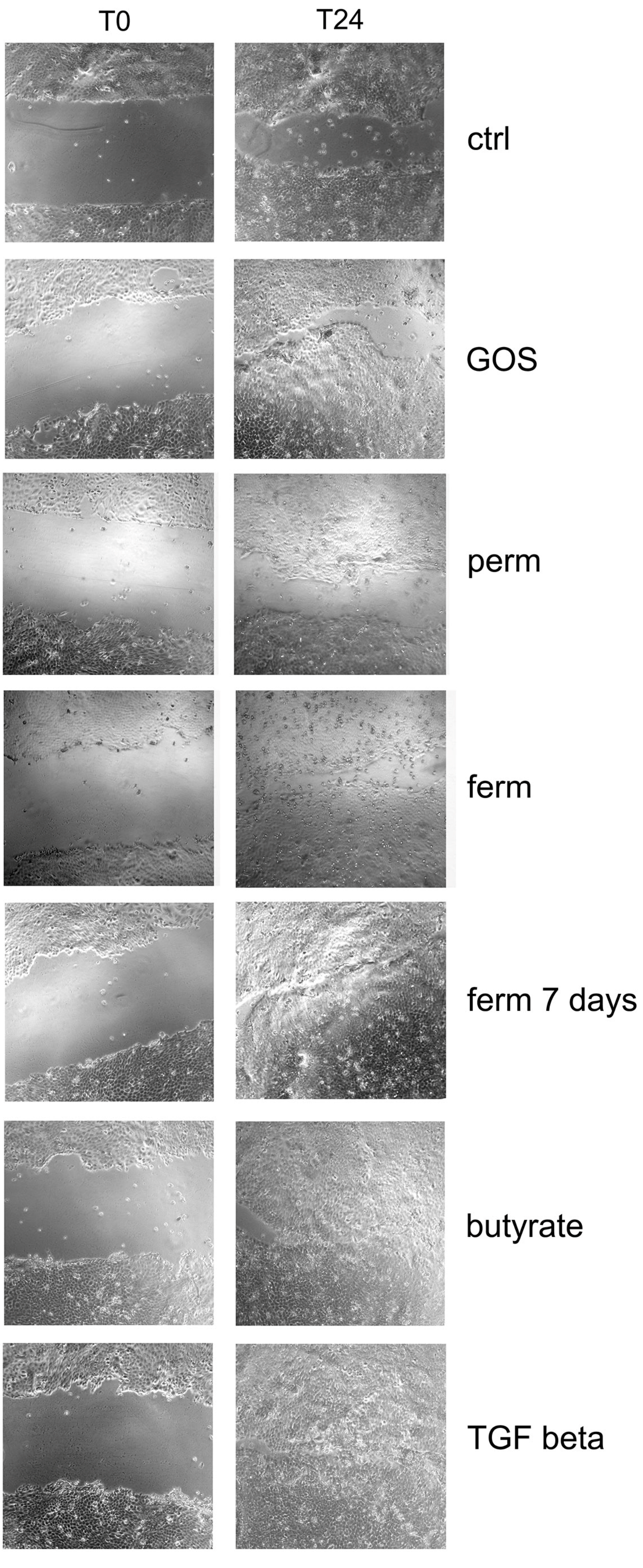

Figure S2. Pictures of cells used for wound healing assay were taken after scratch (T0) and after 24 h of treatment (T24). ctrl, control; perm, permeate; ferm, fermented product; GOS, galactooligosaccharides; TGF, transforming growth factor.

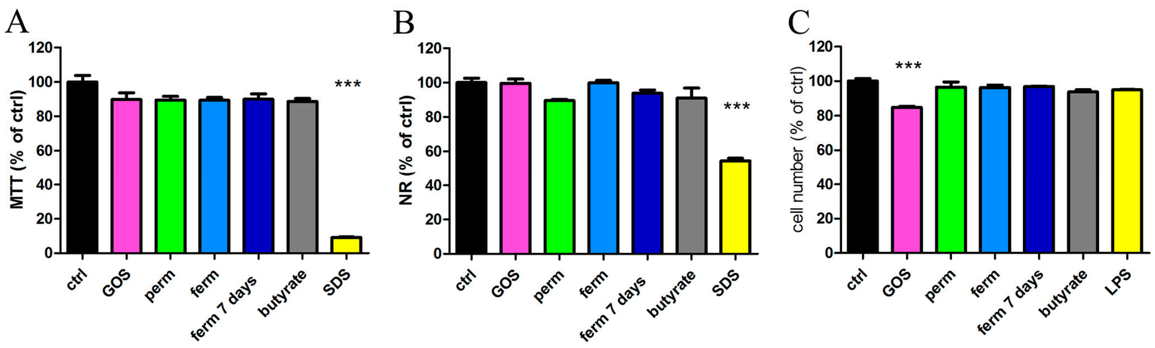

Figure S3. GOS and whey derivatives do not affect the viability and proliferation of HaCaT cells. After two days of treatment, cells were analyzed by MTT (**A**) and neutral red assay (**B**) to exclude cytotoxicity, and proliferation was assessed by crystal violet staining (**C**). Positive controls for toxicity is represented by 0.002% SDS. The data represent the means ± SEM of three independent experiments. *** $p < 0.001$ compared with the control. ctrl, control; perm, permeate; ferm, fermented product; GOS, galactooligosaccharides; SDS, Sodium dodecyl sulfate; LPS, lipopolysaccharide from *Escherichia coli* O111:B4.
